# Bat lyssavirus should be further monitored in Rondônia state, Brazil

**DOI:** 10.1590/0037-8682-0420-2019

**Published:** 2020-04-22

**Authors:** Lifeng Zhao, Teng Chen, Faming Miao, Junfeng Li, Haijun Du, Jinghui Zhao

**Affiliations:** 1 College of Animal Veterinary Medicine, Jilin Agricultural Science and Technology University, Jilin 132101, China.; 2 Key Laboratory of Jilin Province for Zoonoses Prevention and Control, Laboratory of Epidemiology, Institute of Military Veterinary Medicine, Academy of Military Medical Sciences, Changchun 130122, China.; 3 Jilin Agricultural University, Changchun 130118, China.; 4 Changchun Sci-Tech University, 1699 Donghua street, Shuangyang District, Changchun 130600, China.; 5 Xuzhou Medical University, Xuzhou 221004, China.

**Dear Editor:**

The article entitled “Rabies virus monitoring in bat populations in Rondônia state, Brazil” is very interesting^1^. Almeida et al^1^ conducted a molecular and serological assessment for the presence of the rabies virus in bats in the area surrounding the Jirau hydroelectric power plant in Rondônia state, Brazil. They found that all 1,183 bat brains analyzed were negative for the presence of rabies virus, and antibodies were noted in 17.5% of 1,049 bats. Environmental changes can indicate an increased risk of a rabies outbreak and circulation in bats. Despite its strengths, the study contains a technical issue that needs to be further addressed.

This study focused on rabies virus, and bat brain and blood samples were collected between 2010 and 2015. Bat brain and blood samples were valuable and rare in terms of scientific significance; thus, the lyssavirus should be further monitored in Brazil. According to the study, “Rabies diagnosis was made using a fluorescent antibody test FAT,” only one test was used to assess the presence of the rabies virus. Heminested PCR assays should also be used for the detection of six genotypes of rabies and rabies-related viruses^2^ ^-^ ^4^. A similar study of lyssavirus was conducted in bats in Croatia and Southeastern Europe^5^.

Rabies viruses belong to the genus *Lyssavirus*. Base on the literature, 16 *Lyssavirus* species, excluding Ikoma lyssavirus and Mokola lyssavirus, have been reported in bats^6^ ^,^ ^7^. Kotalahti bat lyssavirusand Taiwan bat lyssavirus were recently isolated from bats^8^ ^,^ ^9^, thus indicating that lyssaviruses are distributed across the world. In Brazil, bat rabies surveillance has been performed in several different regions and bat species; however, no study on bat lyssavirus can be found in the literature. Therefore, we strongly advise that the surveillance of bat lyssavirus should be performed in Brazil.
